# Macrophage Inhibitory Factor-1 (MIF-1) controls the plasticity of multiple myeloma tumor cells

**DOI:** 10.1371/journal.pone.0206368

**Published:** 2018-11-01

**Authors:** Danielle Joseph, Jason P. Gonsky, Stacy W. Blain

**Affiliations:** 1 School of Graduate Studies, SUNY Downstate Medical Center, Brooklyn, N.Y., United States of America; 2 Department of Medicine, Division of Hematology and Oncology, Kings County Hospital Center and SUNY Downstate Medical Center, Brooklyn, N.Y., United States of America; 3 Depts. of Pediatrics and Cell Biology, SUNY Downstate Medical Center, Brooklyn, N.Y., United States of America; University of Catanzaro, ITALY

## Abstract

Multiple Myeloma (MM) is the second most common hematological malignancy with a median survival of 5–10 years. While current treatments initially cause remission, relapse almost always occurs, leading to the hypothesis that a chemotherapy-resistant cancer stem cell (CSC) remains dormant, and undergoes self-renewal and differentiation to reestablish disease. Our finding is that the mature cancer cell (CD138+, rapidly proliferating and chemosensitive) has developmental plasticity; namely, the ability to dedifferentiate back into its own chemoresistant CSC progenitor, the CD138–, quiescent pre-plasma cell. We observe multiple cycles of differentiation and dedifferentiation in the absence of niche or supportive accessory cells, suggesting that soluble cytokines secreted by the MM cells themselves are responsible for this bidirectional interconversion and that stemness and chemoresistance are dynamic characteristics that can be acquired or lost and thus may be targetable. By examining cytokine secretion of CD138- and CD138+ RPMI-8226 cells, we identified that concomitant with interconversion, Macrophage Migration Inhibitory Factor (MIF-1) is secreted. The addition of a small molecule MIF-1 inhibitor (4-IPP) or MIF-1 neutralizing antibodies to CD138+ cells accelerated dedifferentiation back into the CD138- progenitor, while addition of recombinant MIF-1 drove cells towards CD138+ differentiation. A similar increase in the CD138- population is seen when MM tumor cells isolated from primary bone marrow aspirates are cultured in the presence of 4-IPP. As the CD138+ MM cell is chemosensitive, targeting MIF-1 and/or the pathways that it regulates could be a viable way to modulate stemness and chemosensitivity, which could in turn transform the treatment of MM.

## Introduction

Multiple myeloma (MM) is the second most prevalent hematological malignancy in the United States, with a survival rate of 47% within the first five years post diagnosis [1,2]. It is characterized by the proliferation of clonal plasma cells within the bone marrow, which can interfere with normal blood cell production and also secrete non-functioning monoclonal antibodies called M proteins [3–5]. The tumor cells colonize the bone marrow, and symptomatically, this results in anemia, lytic bone lesions, and hypercalcemia, in addition to renal failure [6,7]. Together with autologous stem cell transplantation and the introduction of novel therapies such as proteasome inhibitors and immunomodulatory drugs, patients frequently show improvement, even to the point of remission. However, relapse is usually occurs, suggesting that there exists a subset of MM tumor cells that are able to evade cytotoxic insult and eventually recapitulate tumor burden [8–10]. This has lead to the hypothesis that a population of chemoresistant cancer stem cells (CSC) persists and remains dormant until a time post-treatment when they reinitiate growth and re-establish tumor burden [10–12].

In addition to cell surface markers, which have been used to identify CSC populations, “stemness” can be phenotypically defined. Similar to the hematopoietic stem cell (HSC), the CSC: 1) exists in a quiescent, slowly proliferating state, 2) possesses the ability to differentiate into more mature progeny, 3) is resistant to chemotherapy, and 4) has the ability to undergo long-term self-renewal in order to maintain the CSC population itself [13–15]. The CSC may be derived from a hematopoietic stem cell that has acquired oncogenic mutation(s) allowing it to become tumorigenic while maintaining stemness OR alternatively from a differentiated tumor plasma cell that dedifferentiates and reacquires stemness properties (i.e. self-renewal, differentiation, quiescence, chemoresistance). MM tumor cells are clonal plasma cells, which have isotype switched, suggesting that the progenitor arises from a post-germinal center B cell lineage, such as a plasma cell precursor or plasma cell itself [5,16]. A marker distinguishing an immature plasma cell (prePC) and a mature plasma cell [17] is the acquisition of CD138 and it is generally believed that the MM CSC population is CD138- as are pre-PCs. Matsui, et al. found that both primary tumor cells isolated from MM patients and MM cell lines are composed of a heterogeneous mixture of CD138- and CD138+ populations [4,16], but only the CD138- cells were able to engraft in the bone marrow of NOD/SCID (non-obese diabetic/ severe compromised immunodeficient) mice [18]. Upon analysis of bone marrow aspirates from these mice, they found that the cells were all CD138+ and expressed the same light chain restriction as the initial patient material, suggesting that the CD138- cells were the CSC population that with time gave rise to the differentiated CD138+ cells. Other laboratories, however, have shown that both CD138- and CD138+ cells can engraft the bone marrow when a different mouse model, NOD/SCID γ (NSG), is used [17], and the authors suggested that the recipient bone marrow niche might play a role in altering stemness. Chaidos et al. showed that patient-derived purified CD138+ plasma cells could produce tumors in animals, but they detected both CD138- and CD138+ cells in the BM of mice, suggesting that the precursor CD138- cells were derived from or were the progeny of the CD138+ as a result of dedifferentiation [17]. These findings imply that a functional plasticity between differentiated progeny and dedifferentiated precursor cells might exist and reconcile the difficulties in clearly defining the CSC population. If interconversion provides a way to continually renew the chemoresistant CSC population, this would contribute to the eventual relapse seen in this disease.

To investigate this, we examined multiple MM cell lines to identify and isolate the CSC population. Our finding is that the ***mature*** myeloma cell (CD138+, rapidly proliferating and chemosensitive) has developmental plasticity; namely, the ability to dedifferentiate back into its own chemoresistant CSC progenitor, the CD138^–^, quiescent plasma cell that possesses stemness potential. As these cells are grown in the absence of accessory cells, this suggests that soluble cytokines secreted by the MM cells themselves are responsible for this bidirectional interconversion. We identified MIF-1 (macrophage migration inhibitory factor-1) as a secreted cytokine that controls this interconversion in MM cell lines, and show that by manipulating the activity or levels of MIF-1 present in the culture media we can shift the set point towards more differentiation or more dedifferentiation.

## Results

### CD138- cells have the characteristics of stemness

**Fig 1:**
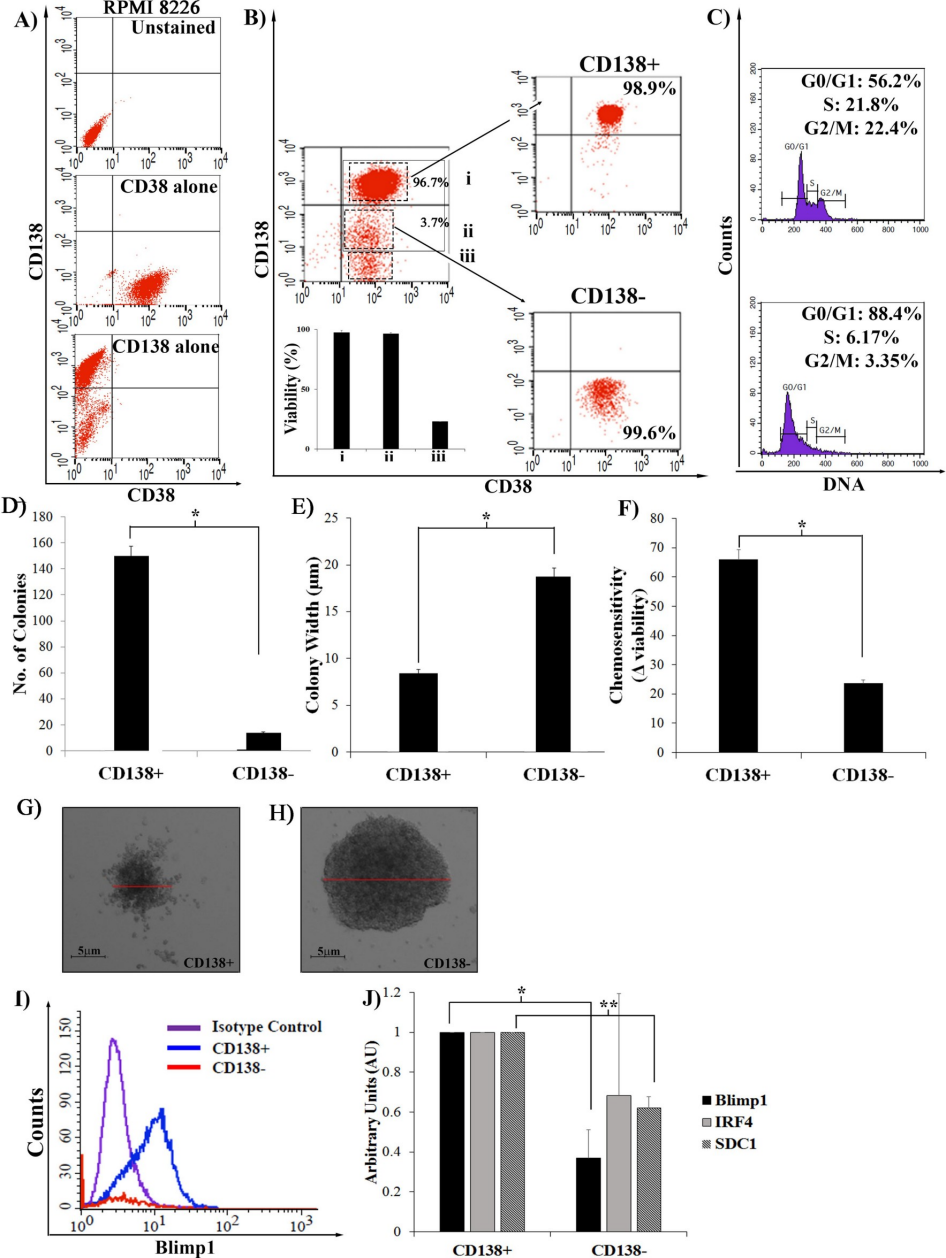
Stemness of RPMI 8226 cells. **A)** Cells analyzed by flow cytometry as unstained, CD38 or CD138 single stained populations to establish gates for double-stained populations. **B)** Cells co-stained with CD138 and CD38, and sorted into CD38+CD138+ (i), CD38+CD138- (ii), CD38+CD138null (iii) populations with high efficiency. **(*Inset*)** Viability of each population, as measured by trypan blue staining and plotted as viability seen in population i (CD38+CD138+). **C)** Each sorted, pure population was independently fixed in ethanol and stained with Vybrant Green and analyzed using CellQuest software to measure cell cycle status. **D,E)** CD138+ and CD138- sorted cells were plated in methylcellulose for 14 days, colonies were counted and widths measured. (n = 4) *p<0.001, **F)** Each sorted population was treated with 0.01μM Bortezomib for 24 h and chemosensitivity measured as the difference in viability (trypan blue) before and after treatment. (n = 4) *p<0.001. **G,H)** Representative colonies formed within methylcellulose by CD138+ and CD138- sorted cells (10^4^cells/ml). **I)** Intracellular staining of Blimp1 expression in sorted CD138+ (blue) and CD138- (red) cells as measured by flow cytometry, isotype control (purple). **J)** RT-PCR to measure the relative levels of Blimp1 (n = 4) and IRF4 (n = 3) and SDC1 (CD138) (n = 3). Levels in CD138+ set to 100, plotted as AU. *p<0.05, **p<0.001, Student t-test. Error bars, mean ± SD.

RPMI 8226 cells were stained with CD138 and CD38 antibodies, and sorted by flow cytometry. Unstained populations and single staining were used to identify doubly stained populations (Fig 1A). Three distinct populations were consistently detected (Fig 1B, dashed boxes, i-iii, panel A in S1 Fig). Each was recovered by sorting with high efficiency, and only population i (hereafter referred to as CD138+CD38+) and ii (CD138-CD38+) were shown to be viable (Fig 1B, inset). The non-viable population iii was gated out of all future analysis. The CD138+ and CD138- populations (i and ii) were consistently detected in a ~9:1 ratio: ~90% of cells were CD138+ and ~10% of cells were CD138- (Fig 1B). The viability of either the CD138+ or CD138- cells does not change with time, as measured by staining at time points post plating of pure populations (panels E,F in S1 Fig). Previous studies have suggested that these CD138- cells were the MM CSC population, and we analyzed these pure, sorted populations to further characterize “stemness” by assessing: cell cycle phase (Fig 1C), long-term self-renewal/clonogenic potential (Fig 1D, 1E, 1G and 1H), chemoresistance (Fig 1F) and expression of differentiation specific markers (Fig 1I and 1J). Purified CD138- and CD138+ populations were stained with Vybrant Green to measure DNA content (Fig 1C). The CD138^+^CD38^+^ cells were rapidly proliferating with only 56% in G0/G1 phase, and 20% and 24% of cells in S and G2/M phase, respectively (Fig 1C, top). In contrast, 88% of the CD138^-^CD38^+^ cells were present in G0/G1 phase, demonstrating that these cells were quiescent or only very slowly proliferating (Fig 1C, bottom). The shift to the left seen in the CD138- cells in the presence of Vybrant Green suggests a more tightly compacted DNA, suggesting that these cells may be in G0 rather than G1 phase. To test the self-renewal capacity of each population, 10^4^ sorted CD138^+^ or CD138^-^ cells were plated in methylcellulose and the formation of colonies was assessed after 14 days. CD138^+^ cells were able to form significantly more colonies than CD138^-^ cells, suggesting that more cells were able to initiate proliferation (Fig 1D). However, these colonies were smaller than those derived from the CD138^-^ precursors (Fig 1E, 1G and 1H). This suggests that fewer CD138- cells entered cycle, but those that did were able to undergo more duplication events than the CD138+ cells, resulting in larger colony width, suggesting that they could proliferate long after the CD138+ cells exhausted their ability to do so. To compare chemotherapeutic response, sorted CD138+ or CD138- populations were treated and mock treated for 24 h. with 0.01μM Bortezomib, a proteasome inhibitor used as frontline therapy in MM treatment. Viability was measured by trypan blue exclusion and chemosensitivity was defined as the difference in viability between mock and Bortezomib treated cells (Fig 1F). CD138+ cells were more sensitive, with a greater loss of viability seen with Bortezomib treatment, than the CD138- cells, where Bortezomib had a reduced effect. Finally, we measured the expression of two transcription factors, Blimp1 and IRF4, which have been shown to be responsible for the differentiation of B cells into plasma cells [3,19,20]. CD138+ cells have higher expression of Blimp1, as detected by intracellular staining (Fig 1I, blue) and RT-PCR analysis (Fig 1J). IRF4 is also expressed preferentially in the CD138+ cells (Fig 1J), suggesting that these are the differentiated progeny, and that the CD138- cells are more immature. CD138 expression is regulated at least in part at the RNA level, as reduced expression of CD138 (SDC1) is detected in the CD138- cells (Fig 1J), although we can’t rule out post-transcriptional regulation. Together, these experiments suggest that the CD138- cells meet the qualifications of stemness: they are quiescent, chemoresistant, able to undergo long term proliferation and do not express the differentiated cell specific markers, IRF4 and Blimp1.

### Cells undergo interconversion, exhibiting plasticity

**Fig 2:**
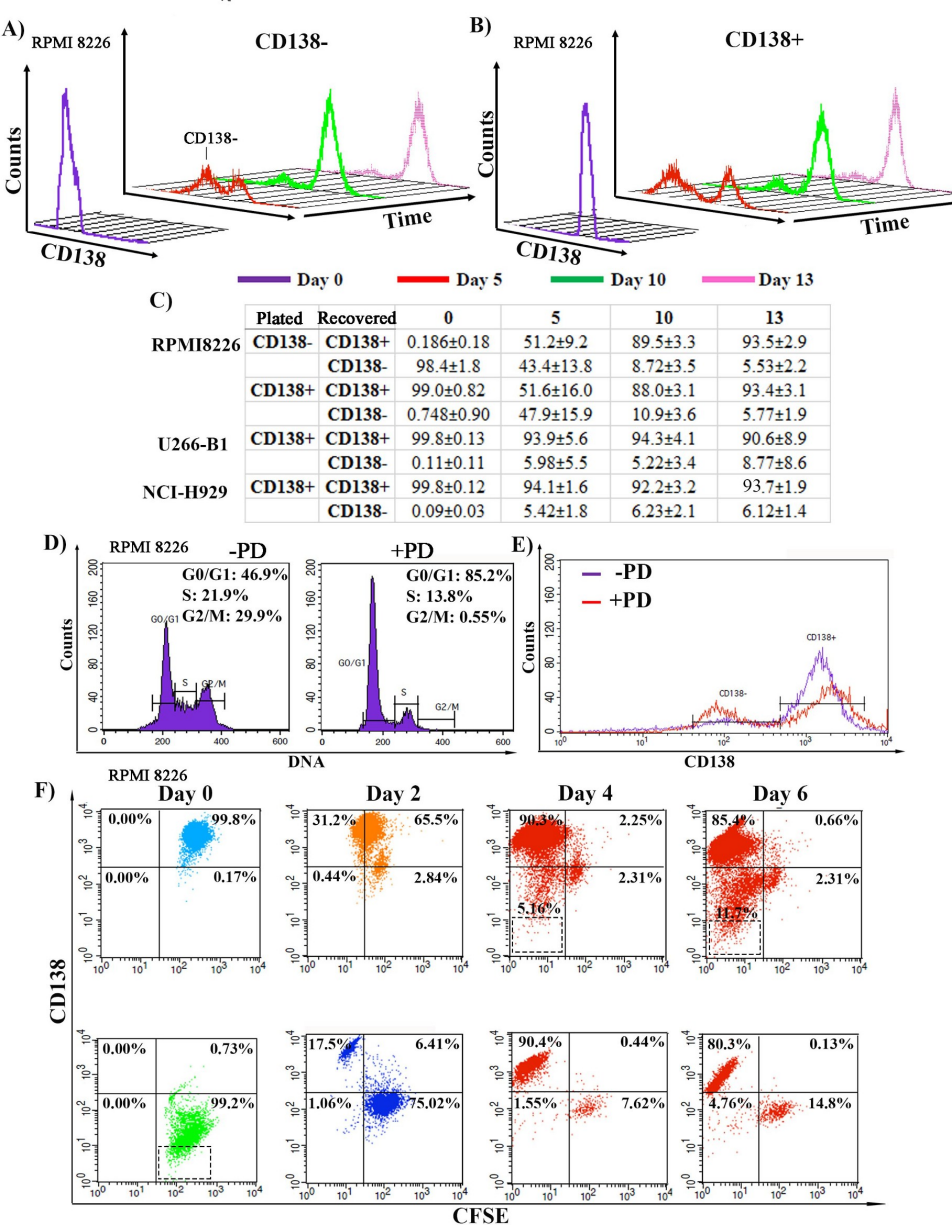
Plasticity of cells. **A, B)** Sorted CD138-CD38+ and CD138+CD38+ populations were plated, aliquots (10,000 cells) were analyzed for CD138 expression at days 5, 10, and 13. Purple line is CD138 content at time of plating (Representative of N = 5). Flow cytometry will not measure expansion of the cultures. **C)** Sorted CD138-CD38+ and CD138+CD38+ cells from U266-B1 and NCI-H929 MM lines plated and analyzed for CD138 status at days 5, 10 and 13. (N = 3, mean+/-SD) **D)** Cell Cycle status of RPMI8226 cells before (left) and after (right) treatment with 5μM PD0332991 (PD). **E)** CD138 expression of RPMI8226 cells before (purple) and after (red) PD treatment for 3 days. **F)** Cells were treated with 5μM PD for 3 days, stained with CFSE, washed, and then followed in culture for 6 days. Aliquots were analyzed for CFSE and CD138 over this time course. Dashed boxes are of non-viable cells (population iii) were not included in the overall percentages of each quadrant.

An additional characteristic of stemness is the ability to undergo differentiation to produce mature progeny. To examine this, pure CD138+ and CD138- RPMI 8226 populations were re-plated (Fig 2A and 2B) and followed for 13 days. CD138+ cells proliferated faster than CD138- cells (panel A in S2 Fig, an increase of 2.4 for CD138+ cells as compared to 1.5 for CD138- cells during the first 5 days), and no significant loss of viability was seen in the populations over the 13 day time course, suggesting that the changes seen in CD138 expression were not reflective of cell death or dying (panels D,E in S2 Fig). Cells were harvested at different time points, and CD138 expression on 10,000 cells was measured by flow cytometry (Fig 2A, 2B and 2C). Within 5 days post plating, pure CD138^-^ cells were able to differentiate and give rise to differentiated CD138^+^ cells: 50% of the total cells were CD138+ by day 5 (Fig 2A, red). The cells continued to differentiate and by day 13, the bulk of the culture was CD138+, reestablishing the 9:1 ratio of CD138^+^:CD138^-^ seen in unsorted cultures (Fig 2A, pink). The mean +/-SE for three experiments are presented in Fig 2C. However, when pure CD138^+^ cells were re-plated, they gave rise to CD138- cells, suggesting that these cells were able to de-differentiate (Fig 2B). Only 40% of the cells remained CD138+ by day 5 (Fig 2B, red). By day 10, the culture started to shift back and 87% of the cells now were CD138+, and by day 13, the 9:1 ratio of CD138^+^:CD138^-^ cells seen in unsorted cultures had been reestablished (Fig 2B, pink). Given the doubling rate of CD138- cells, the emergence of CD138- cells from the pure CD138+ population cannot be due to a small, undetectable, pre-existing CD138- population emerging. Thus, the CD138- cells could differentiate (Fig 2A), while the CD138+ cells could de-differentiate (Fig 2B), suggesting that the cells could interconvert in culture.

To analyze whether this plasticity was intrinsic to the RPMI8226 cell line, or was a quality of other human MM cell lines, NCI-H929 and U266-B1 cells were sorted into CD138^+^ populations (panels B,C,F-I, J-Q in S2 Fig). Unsorted NCI-H929 and U266-B1 cultures have different set points: instead of a 9:1 ratio seen with RPMI8226 cells, the NCI-H929 and U266-B1 cells have fewer CD138- cells and have ratios of 95:5 CD138+:CD138- cells (panels J-Q in S2 Fig). When pure populations of NCI-H929 and U266-B1 CD138+ cells were re-plated, with time, CD138^-^ cells were detected (Fig 2C). By day 13, the cultures had reestablished the ~9:1 CD138+:CD138- ratio, suggesting that plasticity was a shared characteristic of MM cell lines.

To further investigate the interconversion of the CD138- and CD138+ populations, we synchronized cells to follow their differentiation or dedifferentiation. Unsorted RPMI8226 cells were treated with PD0332991 (Palbociclib), a specific inhibitor of cyclin-dependent kinase 4/6, to arrest cells in G1 phase. After 72 h. of PD0332991 treatment, cell cycle profiles were measured using the DNA stain Vybrant Green, in mock and PD0332991 treated cells, and PD0332991 treatment dramatically increased the G1 phase content, demonstrating arrest (Fig 2D, -PD: 46.9%, +PD, 85.2%). Cells were stained with CD138, and we found that PD0332991 treatment caused a change in the CD138+:CD138- ratio, with an increase in the CD138- pool detected (Fig 2E, red), suggesting that G1 phase arrest and CD138 status might be linked. PD0332991 arrested cells were then subsequently stained with the fluorescent cell permeable dye CFSE. This dye is retained in the cytoplasm, and decreases its fluorescent intensity by half upon each cycle of cell division. This allows us to track cell proliferation as measured by loss of CFSE of both the CD138+ and CD138- populations in tandem with their differentiation status, as measured by CD138 expression. PD0332991 arrested, CFSE stained cells were sorted into two pure populations: CD138^+^CD38^+^CFSE^hi^ and CD138^-^CD38^+^CFSE^hi^ (Fig 2F, Day 0). PD0332991 was removed from the culture through sequential washing with fresh media and the labeled cells were replated. Cells were harvested every two days and analyzed for CFSE and stained for CD138. CD138^-^ cells (bottom panel) were able to divide (reducing CFSE content) and differentiate (increasing CD138 content) and within two days, 17.5% of the population had become CD138^+^CFSE^lo^ (Fig 2F, bottom panel, Day 2). 75% of the population did not divide, and while there was a slight increase in CD138 expression when compared to Day 0, these cells with high CFSE remained within the CD138- gate. By days 4 and 6, the bulk of the culture had divided to become CD138^+^CFSE^lo^, while a small pool of cells remained CFSE+CD138^-^ and had not divided (Fig 2F, bottom panel, Days 4, 6). The small contaminating pool of CD138+ cells (0.73% or 1825 cells) cannot account for the increase seen in CD138+ cells by day 2 (panels R-U in S2 Fig). Given the growth rate of CD138+ cells (1.2/day), we would only have detected 2190 cells at day 2, and instead we detected 76,480 cells or 23.9% of the total population of 320,000 cells. Thus, CD138- cells themselves give rise to CD138+ progeny, and this appears linked to cell division.

When sorted, arrested, CD138^+^ CFSE^hi^ cells were replated and released from arrest (Fig 2F, top panel), by day 2, the bulk of the cells are CFSE^mid^ (between 10^1^−10^2^) but remain CD138+, suggesting that they are starting to divide but maintain the CD138 marker (top quadrants). However, a new population, CD138^mid^CFSE^mid^, is also detected, suggesting that some cells are losing expression of CD138 as they divide. This CD138^mid^CFSE^mid^ population continues to decrease in CD138 expression through the last two time points, becoming CD138^-^CFSE^mid^, without a further decrease in CFSE fluorescence, supporting that they are dividing slowly and dedifferentiating. By day 6, three populations are visible: 85.4% are CD138^+^CFSE^lo^ (CD138+ cells that divided into CD138+ cells); 2.3% are CD138^-^CFSE^mid^ (dedifferentiated CD138- cells that may have divided once but then stopped and has been maintained since day 2); 11.7% are CD138^-^CFSE^lo^ (CD138+ cells that dedifferentiated and then divided into CD138- cells). All of these populations are viable and are outside of the non-viable population iii gate (dashed box) from Fig 1. Again, the small contaminating population of CD138- cells (0.17% or 425 cells) is not responsible for the increase seen in this population (13,2000 cells or 3.3% of the total) (panels R-U in S2 Fig). Thus, this suggests that not only are CD138^+^ cells able to proliferate but are also able to de-differentiate, giving rise to the CD138^-^ precursor.

### Dedifferentiated CD138- cells exhibit characteristics of stemness

**Fig 3:**
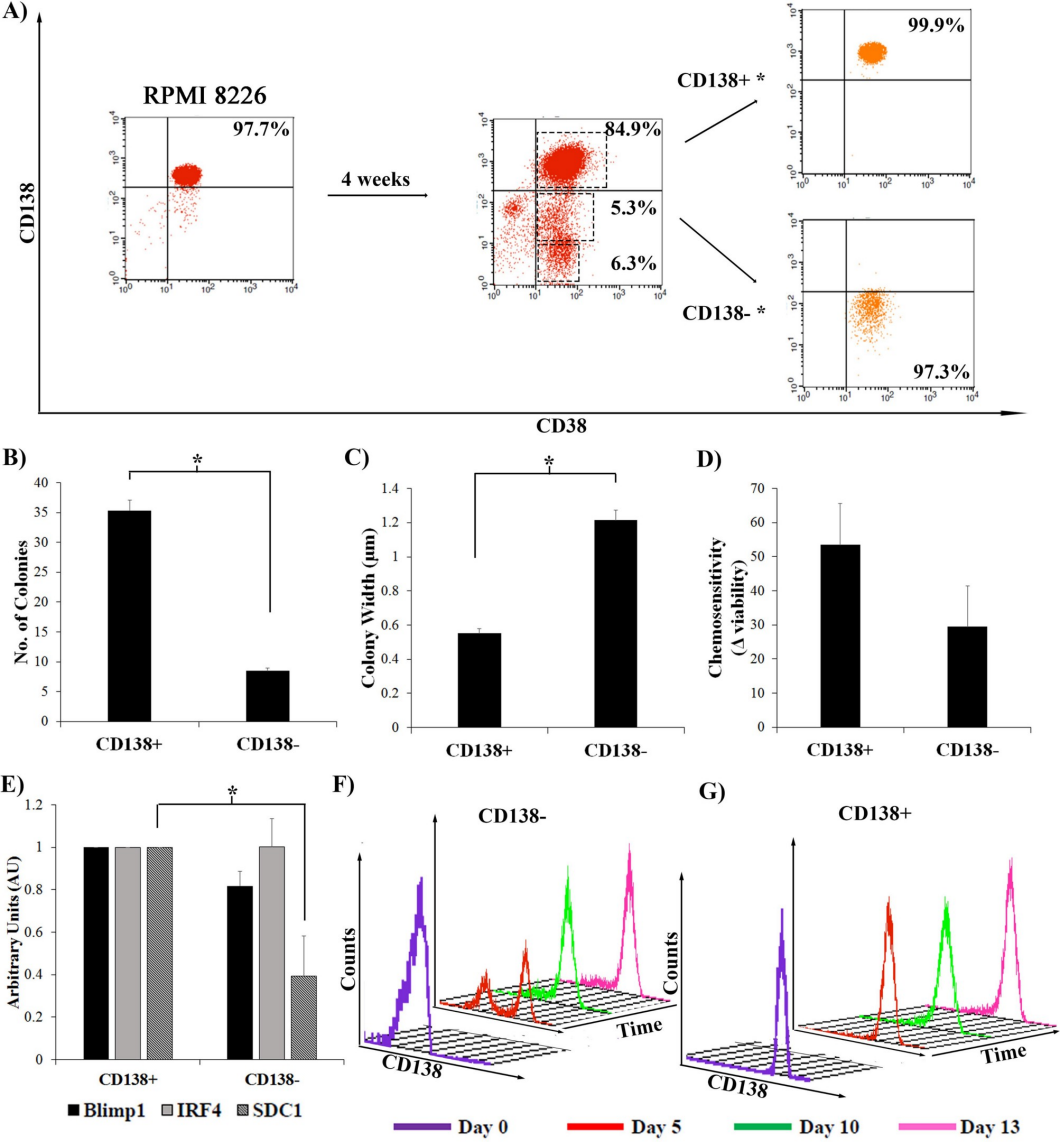
Authenticity of newly dedifferentiated RPMI 8226 CD138- cells. **A)** Sorted CD138+CD38+ populations were plated for four weeks, and then re-sorted based on CD138 status, to yield newly differentiated CD138+* and dedifferentiated CD138-* populations. Representative of N = 3. Dashed boxes is non-viable (population iii). **B,C)** These sorted CD138+* and CD138-* cells were plated in methylcellulose, colonies were counted and measured (N = 3) *p<0.05. **D)** CD138+* and CD138-* cells were plated and treated with Bortezomib for 24 h. Chemosensitivity was measured as the difference in viability before and after treatment (N = 3). **E)** RT-PCR on CD138+* and CD138-* populations to assess the levels of Blimp1, IRF4, and SDC1 (CD138) (N = 3). The levels seen in CD138+ cells was set to 100, plotted as AU, *p<0.05. Error bars, mean ± SD. **F,G)** Sorted CD138+* and CD138-* populations were plated, and analyzed for CD138 by flow cytometry at days 5, 10, and 13. Purple line is CD138 content at time of plating (Representative of N = 3).

To determine whether MM cells could undergo multiple cycles of differentiation and de-differentiation and remain phenotypically the same as CD138- or CD138+ cells isolated from the parent RPMI8226 cell line, pure CD138^+^ cells (S3 Fig) were re-plated and maintained for 4 weeks (Fig 3A). During this time, the 9:1 ratio of CD138^+^:CD138^-^ cells was reestablished (Fig 3A), and the ratio of non-viable population iii was similar, suggesting that significant cell death had not occurred during this time period. Pure populations (CD138^+^* and CD138^-^*) were recovered from these cells and tested by the stemness assays described in Fig 1. CD138^+^* and CD138^-^* cells were plated in methylcellulose and the formation of colonies was assessed. As seen with cells isolated from the parent cell line, the CD138^+^* cells formed significantly more colonies than the CD138-* cells (Fig 3B), but the CD138^-^* population formed significantly larger colonies (Fig 3C). Both populations were treated with Bortezomib or mock treated for 24 h. and similar to cells isolated from the parent line, CD138^+^* cells showed increased sensitivity to chemotherapeutic treatment as indicated by the greater change in viability as compared to the more chemoresistant CD138^-^* cells (Fig 3D). The expression of Blimp1, IRF4 and SDC1 (CD138) in the doubly sorted CD138+* and CD138-* cells was measured by RT-PCR (Fig 3E), and as seen with cells isolated from the parent cell line, CD138^-^* cells had lower levels of Blimp1 and SDC1 RNA (Fig 3E), while IRF4 levels between the populations were more similar (Fig 3E). The doubly sorted CD138^-^* cells were replated and followed in the differentiation assay for up to 13 days (Fig 3F). These cells were able to differentiate again, with kinetics similar to those seen in cells freshly isolated from the parent line, re-establishing the ~9:1 ratio of CD138^+^:CD138^-^ cells by day 13 (Fig 3G). Doubly sorted CD138^+^* cells were able to de-differentiate by day 5 and re-establish the ~9:1 ratio of CD138^+^:CD138^-^ sorted cells by day 13 (Fig 3F). The kinetics of dedifferentiation of the CD138^+^* cells was different in that there was no large CD138^-^ population by day 5 as seen with the parent CD138^+^ cells (Fig 2B). However, in general, both of the doubly sorted populations maintained plasticity and could differentiate and/or dedifferentiate respectively.

### Modulation of MIF-1 activity alters interconversion

**Fig 4:**
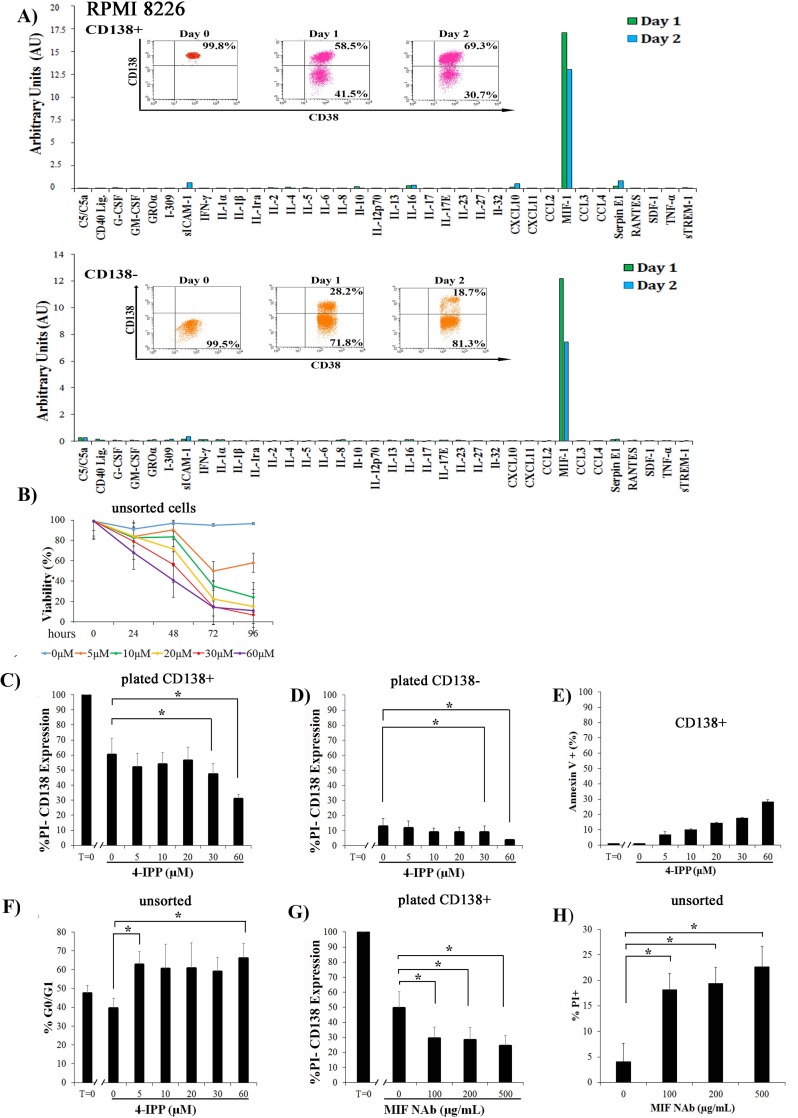
MIF-1 regulates differentiation status of RPMI 8226 cells. **A)** Sorted CD138+ or CD138- cells were plated and media was harvested at T0, T24 and T48 and used in the cytokine array assay. AU plotted as the expression of 36 different cytokines. Representative of N = 3 (CD138+) and N = 2 of (CD138-). Inset: CD138 and CD38 status at each time point, assessed by flow cytometry, to verify differentiation and dedifferentiation of populations during the timecourse. **B)** Viability measured by PI staining of cells treated with different concentrations of 4-IPP for different timepoints. (N = 3). **C)** Sorted populations of CD138+ or **D)** CD138- cells were plated and then treated with increasing concentrations of 4-IPP for 24 h. Cells were stained with CD138 and PI and expression of CD138 in viable (PI negative) cells is plotted (N = 3). T = 0 is CD138 content at time of plating before 4-IPP treatment. **E)** Sorted CD138+ cells were plated, treated with increasing concentrations of 4-IPP for 24 h. and then stained with Annexin V, followed by flow cytometric (N = 3) Test of trend, p = 0.008. **F)** Unsorted cells were plated, treated with increasing concentrations of 4-IPP for 24 h., and then stained with Vybrant Green, followed by cell cycle analysis (N = 3). **G**) Sorted CD138+ cells were plated, treated with increasing concentrations of MIF Nab for 24 h. Cells were stained with CD138 and PI and expression of CD138 in viable (PI negative) cells is plotted (N = 3). T = 0 is CD138 content at time of plating. **H)** Unsorted cells were plated, treated with increasing concentrations of MIF NAb for 24 h. Viability was measured by PI staining (N = 3). *p<0.05. Error bars, mean ± SD.

While interconversion of MM cells has been reported before [17,21], this is the first time that plasticity has been reported to occur in 2D culture in the absence of accessory cells. This suggests that the MM cells themselves controlled their differentiation or dedifferentiation status and we hypothesized that the cells might secrete cytokines into the culture media that influence interconversion. To examine cytokine secretion directly, CD138+ and CD138- cells were sorted, plated, and conditioned media was harvested at days 1 and 2, and a cytokine array was used to detect the presence of 36 cytokines simultaneously (Fig 4A). The cytokine values detected at time 0 (plating) and in the media alone were subtracted to generate the change in expression, and resultant levels were plotted as AU (Fig 4C, S4 Fig). While several different cytokines were modestly increased in supernatant from both CD138+ and CD138- cells, the expression of Macrophage Inhibitory Factor 1 (MIF-1) increased rapidly and significantly in both populations. MIF-1 is an inflammatory cytokine, secreted by many cell types, and shown to increase survival and proliferation of several stem cell lineages [22–25]. We hypothesized that the burst of MIF-1 secreted into the media might promote interconversion of the individual populations, and that blocking MIF-1 activity with 4-IPP, a small molecule inhibitor of MIF-1, might alter the kinetics of dedifferentiation or differentiation. Pure populations of CD138+ cells were treated with different concentrations of 4-IPP and viability was followed by PI staining (Fig 4B) Because treatment with 4-IPP decreased cell viability with time and concentration, we only analyzed viable cells, as measured by PI staining, for CD138 status in future experiments (Fig 4C, 4D, 4G and 4H). When pure CD138+ cells were plated and then mock treated, roughly 60% of CD138+ cells remained CD138+ at 24 h., suggesting that 40% had dedifferentiated during this time frame (Fig 4C, compare T = 0 to [0]). However, when treated with 30 or 60 micromolar 4-IPP, only 50 or 35% remained CD138+ respectively, suggesting that blocking MIF-1 activity increased dedifferentiation (50 to 65% became CD138- during the same time period). When CD138^-^ populations were plated and treated for 24 h. with 4-IPP, 12% differentiated (88% remained CD138-) (Fig 4D, T = 0 to [0] 4-IPP). However, in the presence of 30 or 60 micromolar 4-IPP, differentiation was slowed, and only 8 and 4% of the PI- cells became CD138+ (92 and 96% of cells remained CD138-) (Fig 4D). This suggests that blocking MIF-1 increased the pool of dedifferentiated cells.

The loss of viability seen in the CD138+ cells in the presence of 4-IPP (Fig 4B) was due to apoptosis, as measured by increased Annexin V staining after 24 h with increasing concentrations of drug (Fig 4E). Annexin V staining of untreated CD138+ cells did not change by the 24 h. timepoint, demonstrating the stability of untreated cells. Treatment of unsorted RPMI8226 cells with increasing concentrations of 4-IPP also caused a significant increase in the G0/G1 phase, as measured by Vybrant Green staining (Fig 4F). While unsorted cells had a G0/G1 content of ~50%, 4-IPP treatment increased this to ~ 60%. As 4-IPP promotes increases in the CD138- pool, this increase in quiescence is consistent.

To validate the specificity of 4-IPP to promote the dedifferentiation of CD138^+^ cells by targeting MIF-1, we used a monoclonal neutralizing antibody (NAb) to block MIF-1 (Fig 4G). Increasing concentrations of MIF NAb were added to pure sorted CD138+ cells for 24 h., and then viable cells, as measured by PI staining, were analyzed for CD138 status (Fig 4G). While approximately 50% of the mock treated CD138+ cells remained CD138+ (50% had dedifferentiated), only 30% of the NAb treated cells remained CD138+ (70% dedifferentiated into CD138- cells within the same time frame). Just like 4-IPP treatment, the viability of the NAb treated cells was reduced. Pure CD138+ cells were plated in the absence or increasing concentrations of MIF NAb for 24 h, stained with PI, and analyzed by flow cytometry (Fig 4H). A similar 20% decrease in viability was seen with NAb addition, suggesting that death might be a physiological response to forced, excessive dedifferentiation or that MIF-1 is a survival factor.

### 4-IPP or NAb treated cells behave like dedifferentiated CD138- cells

**Fig 5:**
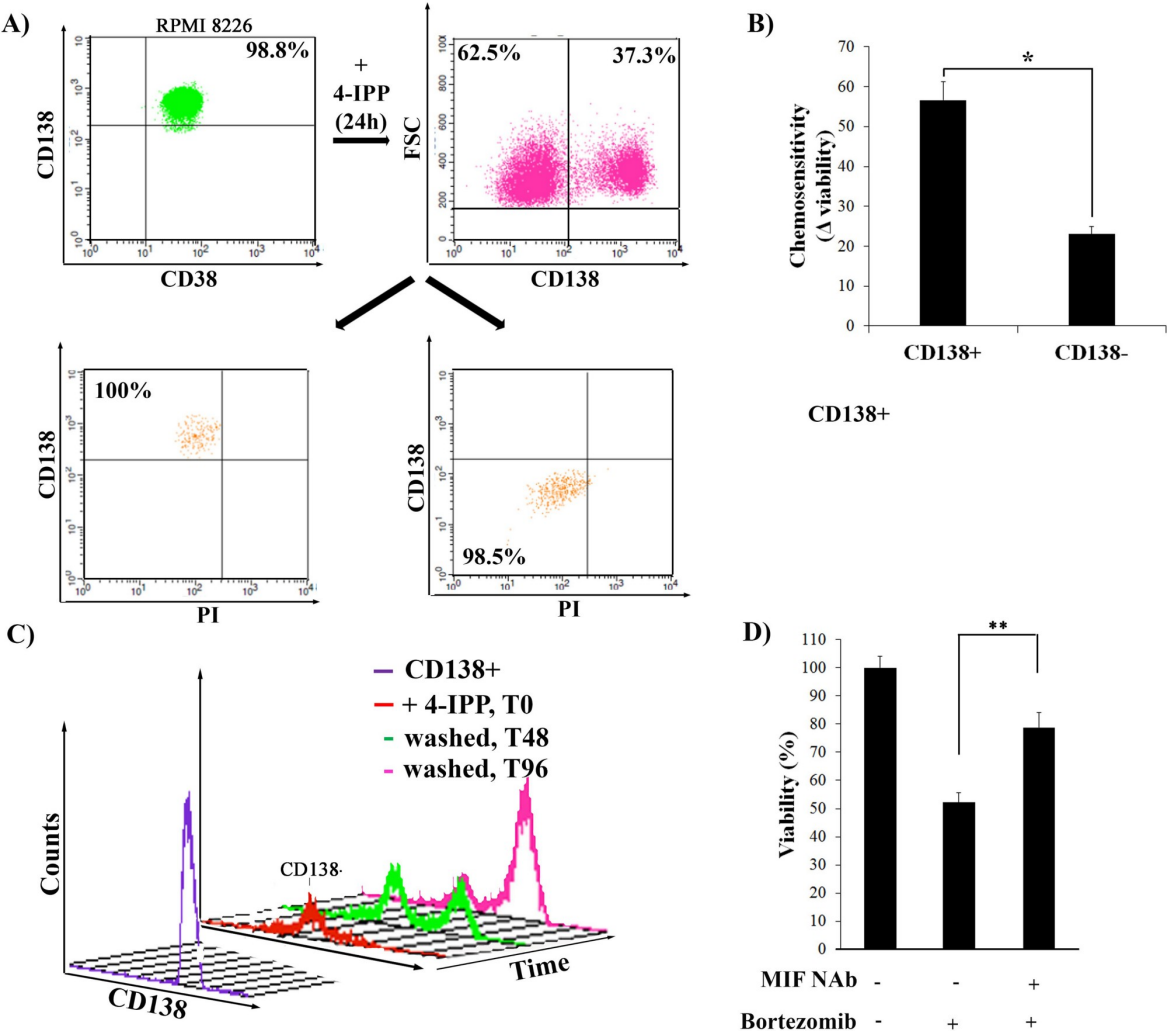
4-IPP dedifferentiated RPMI 8226 CD138- cells are chemoresistant. **A)** Sorted CD138+ cells were treated with 60μM 4-IPP for 24 h. Cells were stained for CD138 and PI and sorted to recover viable (PI negative) CD138+ and CD138- cells. **B)** These sorted cells were plated and treated with Bortezomib for 24 h and chemosensitivity was measured as the difference in viability before and after treatment by trypan blue exclusion. *p<0.01 **C)** Sorted CD138+ cells were treated with 60μM 4-IPP for 24 h. 4-IPP was then washed out and cells were re-plated, harvested at different timepoints, and analyzed for CD138 expression. Purple line is CD138 content at time of plating, demonstrating purity of the initially plated population. Red line is CD138 content in cells treated with 4-IPP for 24 h. CD138 expression measured at 48 (green) and 96 h (pink) post 4-IPP wash out. **D)** Sorted CD138+ cells were plated and untreated (lane 1), treated with Bortezomib. (lane 2), or treated + MIF Nab and Bortezomib for 24 h (lane 3). The MTT assay used to assess viability of the different populations, mean absorbance values of untreated cells (lane 1) was set to 100 (N = 3). **p<0.001 t-test with Satterthwaite corrections to allow for possible unequal variances. Error bars, mean ± SD.

Blocking MIF-1 activity appeared to drive or maintain dedifferentiation status in both the CD138+ and CD138- populations (Fig 4C–4H) and we wanted to determine whether CD138- and CD138+ cells generated by blocking MIF-1 activity behaved like untreated CD138- and CD138+ cells, in terms of chemotherapeutic response. Sorted parent CD138^+^ cells were treated with 60 μM 4-IPP for 24 h to promote dedifferentiation, and 62.5% of these cells became CD138- (Fig 5A). These cells were doubly stained with CD138 and PI to isolate viable CD138^+^ and CD138^-^ populations (Fig 5A, right), which were then mock treated or treated with Bortezomib for 24 h. Chemosensitivity was measured as the difference in viability between the mock and Bortezomib treated populations (Fig 5B). The 4-IPP derived CD138- cells were significantly less sensitive to chemotherapeutic treatment than the CD138+ cells, similar to what was seen with the parent derived CD138- populations (Fig1F). CD138+ cells treated with 4-IPP for 48 h. (to become CD138-) (Fig 5C, red), were replated without drug and analyzed for CD138 expression at 48 (green) and 96 (pink) h. Whereas 62% of 4-IPP treated cells were CD138- (red) at the time of replating, by 48 h. without drug, a large CD138+ population appeared, and by 96 h, 73% were CD138+. Thus, the CD138- cells derived by 4-IPP treatment could recover and regained characteristics of stemness: they were chemoresistant and could differentiate.

MIF NAb treatment also increased the pool of CD138- cells (Fig 4G) and we wanted to determine whether this treatment also increased chemoresistance in these CD138- cells (Fig 5D). Sorted CD138^+^ were treated +/- NAB for 24 h., and then were treated with Bortezomib for an additional 24 h to measure viability using the MTT assay. CD138+ cells are sensitive to Bortezomib, and their viability decreases to 48% following treatment (lane 2). CD138+ cells pre-treated with NAb, however, were less sensitive to Bortezomib and their viability was only reduced 17% with treatment (lane 4). Consistent with what was seen with 4-IPP treatment, CD138+ cells treated with MIF NAb were more chemoresistant than untreated cells, consistent with the increase in CD138- populations.

#### rMIF promotes differentiation into CD138^+^ cells

**Fig 6:**
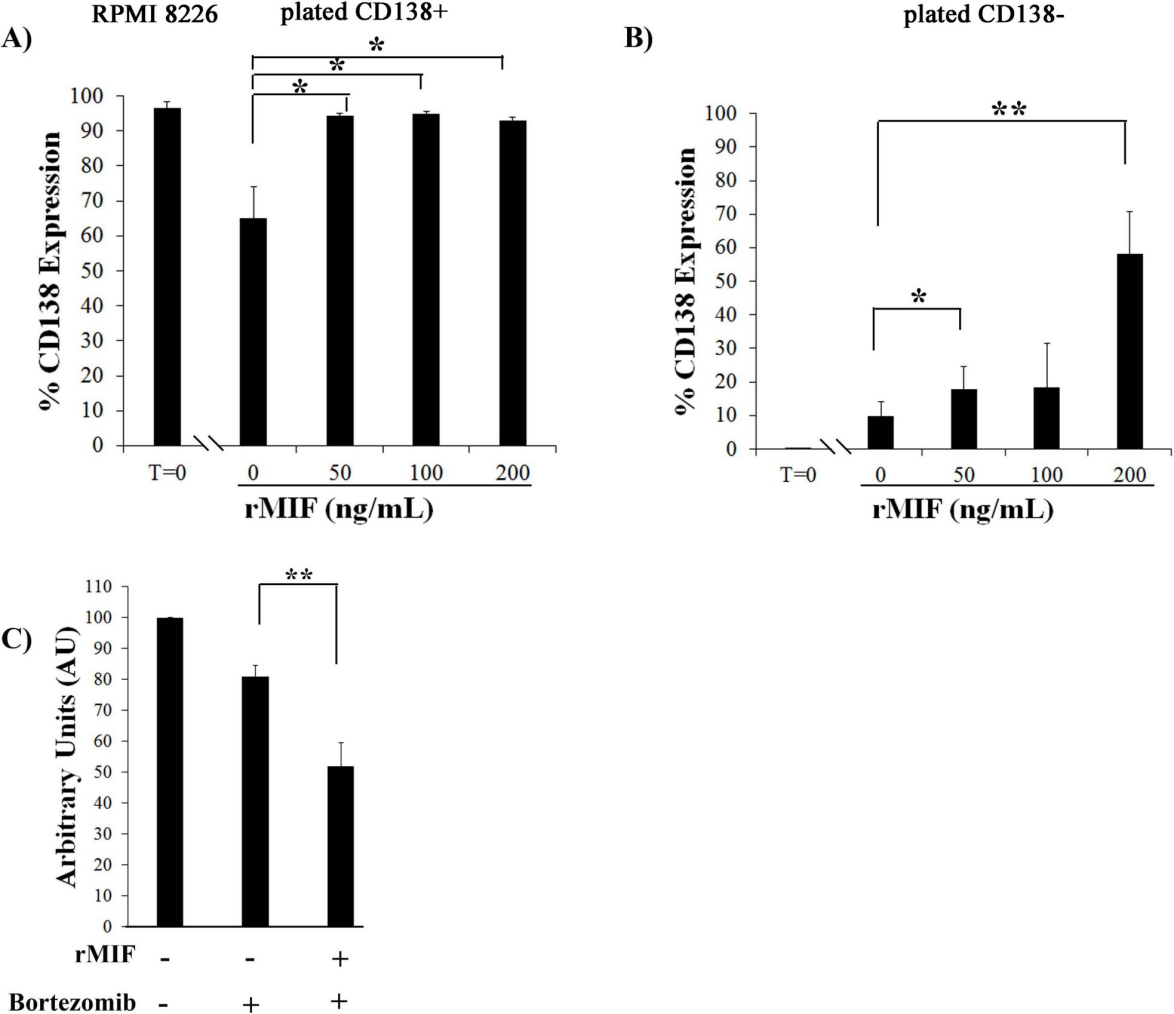
rMIF promotes the differentiation and maintenance of RPMI 8226 CD138+ cells. **A)** Sorted CD138+ and **B)** CD138- cells were plated, treated with increasing concentrations of rMIF for 24 h. Cells were stained with CD138 and PI and the CD138 content of viable (PI negative) cells is plotted (N = 3). T = 0 is CD138 content at time of plating. **C)** Sorted CD138- cells were plated and untreated (lane 1), treated with Bortezomib. (lane 2), or treated + rMIF and Bortezomib for 24 h (lane 3). The MTT assay used to assess viability of the different populations, mean absorbance values of untreated cells (lane 1) was set to 100 (N = 3). **p<0.001 t-test with Satterthwaite corrections to allow for possible unequal variances. Error bars, mean ± SD.

Because blocking MIF-1 activity with either 4-IPP or MIF NAb promoted dedifferentiation, we hypothesized that an excess of rMIF might promote differentiation into CD138+ cells. Sorted CD138^+^ cells were treated with rMIF at increasing concentrations, and PI and CD138 status was measured after 24 h (Fig 6A). Cell viability was not affected by rMIF treatment. While approximately 60% of mock treated CD138+ cells remained CD138+ (40% had dedifferentiated to CD138- cells, Fig 6A, compare T = 0 to [0]), in the presence of 50 ng/ml rMIF, almost 90% of cells remained CD138+, suggesting that rMIF prevented dedifferentiation and allowed the maintenance of the CD138^+^ population (Fig 6A, compare T = 0 to [50] rMIF). When sorted, pure CD138^-^ cells were mock treated, 10% of cells differentiated and became CD138+ within 24 h. (90% remained CD138-, Fig 6B, compare T = 0 to [0] rMIF), but when treated with different concentrations of rMIF, an increase in CD138+ cells was also seen. This suggests that the presence of rMIF helped to maintain and/or generate a CD138+ population by promoting differentiation.

To determine whether the CD138+ cells generated by rMIF treatment were more sensitive to chemotherapeutic agents, CD138^-^ cells were treated +/- rMIF for 24 h., and then treated with Bortezomib for 24 h to measure viability in the MTT assay (Fig 6C). CD138- cells are relatively resistant to Bortezomib, and viability of treated cells was only reduced by 20% (lane 2). CD138- cells treated with rMIF, however, were more sensitive to Bortezomib and viability was now reduced 46% by treatment (lane 4).

#### CD138+ cells secrete more MIF-1 than CD138- cells

**Fig 7:**
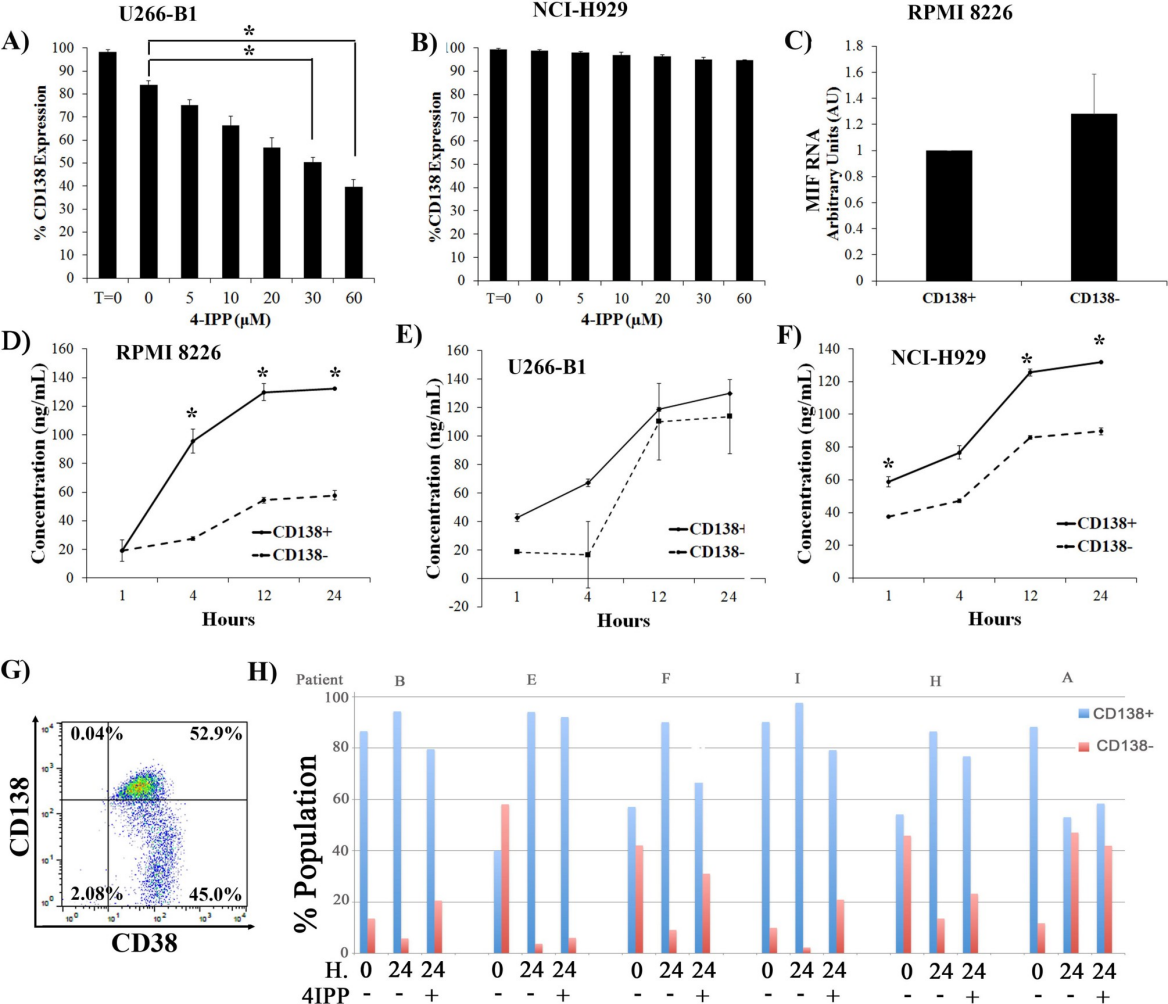
MIF-1 regulates differentiation in other cell lines and primary patient material. **A)** Sorted CD138+ U266 cells and **B)** NCI-H929 cells were plated and treated with increasing concentrations of 4-IPP for 24 h. Cells were then stained with CD138 and PI, and CD138 content of viable (PI negative) cells is plotted. T = 0 is CD138 status at time of plating (N = 3) *p<0.05. **C)** Steady state MIF-1 levels of sorted CD138+ and CD138- RPMI 8226 cells as measured by RT-PCR. **D, E, F)** Sorted CD138+ (solid) and CD138- (dashed) cells from RPMI8226 (D), U266-B1 (E), and NCI-H929 (F) lines were plated and media harvested at 1, 4, 12, and 24 h. post plating for use in the MIF ELISA assay (N = 3). *p<0.001 **G)** MM cells derived from human bone marrow aspirates from patient H, stained for CD138 and CD38 at time of harvest and analyzed by flow cytometry. Data is also plotted in panel H. **H)** BM derived MM cells were placed in culture +/- 4-IPP for 24 h., then were stained with CD38 and CD138. The CD138 status of the CD38+ gate is shown.

We had seen that NCI-H929 and U266-B1 cells also underwent differentiation and dedifferentiation when plated as pure populations (Fig 2C). Similar to the RPMI 8226 line, when U266-B1 CD138+ cells were treated with increasing concentrations of 4-IPP, they also exhibited a marked decrease in the level of CD138+ cells (and an increase in dedifferentiated CD138- cells) (Fig 7A). 4-IPP did not increase dedifferentiation of the NCI-H929 cells within this 48h. timeframe (Fig 7B). Thus, MIF-1 activity appeared to regulate interconversion in at least two MM cell lines. As we had previously seen interconversion after 5 days, 48 hrs may have been too short a timeframe to see an effect of 4-IPP on NCI-H929 cells. However, as the viability of cells exposed to 4-IPP decreases at times greater than 24 h., we could not assay for increased time.

The cytokine array assay had demonstrated that MIF-1 was secreted from both pure CD138- and CD138+ cells (Fig 4A). We examined RNA levels in both CD138+ and CD138- RPMI 8226 populations using RT-PCR and did not find any statistically significant differences (Fig 7C). Using a quantitative MIF-1 ELISA kit, we were able to measure MIF-1 cytokine secretion at 1, 4, 12 and 24 h. post plating of pure populations of all three cell lines (Fig 7D, 7E and 7F). CD138+ cells secreted statistically significant more MIF-1 than CD138- cells in the RPMI8226 and the NCI-H929 cell lines (Fig 7D and 7F), while no significant differences were detected for U266-B1 cells (Fig 7E), although the trends were the same.

#### MIF-1 interconversion in primary MM cells

To examine whether MIF-1 regulates interconversion in vivo, bone marrow aspirates from MM patients were obtained with informed consent (patient characteristics described in S5 Fig). MM tumor cells were purified using the Rosette Step MM purification kit, which removes other contaminating hematopoietic cells that contain CD2, CD14, CD33, CD41, CD45RA, CD66b markers and glycophorin A on red blood cells (RBCs). Viability was always >75% as measured by trypan blue staining. This material was either stained immediately with CD138 and CD38 antibodies (Fig 7G and 7H, T = 0) or plated in tissue culture in the absence or presence of 4-IPP for 24 h. (Fig 7H). The ratio of CD138+:CD138- cells varied among patients, with several patients having very high CD138- content (Fig 7G, patient E, F, H). Variability in the CD138 content is consistent with previous reports [26]. Primary material was replated +/- 4-IPP, cells were harvested at 24 h., stained with CD138 and CD38 antibodies and analyzed by flow cytometry (Fig 7H). Viability as measured by trypan blue staining was unchanged. In 5 out of 6 patients, after 24 h. in culture, cells differentiated into CD138+ (Fig 7H, patients B, E, F, I, H). This was most obvious in the patients with high CD138- content at the time of harvest (Fig 7H, patients E, F, H), suggesting that replating promotes differentiation. However, when plated in the presence of 4-IPP, the CD138- population either increased relative to T = 0 (patients B, I) or did not decrease as significantly as seen in the absence of 4-IPP (patients E, F, H), consistent with the role of 4-IPP on the cell lines. In patient A, plating +/- 4-IPP appeared to increase the CD138- population, and the significance of this is unclear.

## Discussion

While there has been some debate in the field regarding the nature of the MM CSC, our data and that of others strongly suggest that the CD138- cell meets the definition of stemness, based on phenotypic characteristics: quiescence, chemotherapeutic resistance, the ability to self-renew and differentiate. We and others have shown that the CD138- population is consistently detected in MM cell lines and material derived from patient BM, suggesting that these are a mixture of the mature, rapidly dividing CD138+ cells, and immature, slowly proliferating CD138- cells [4,16–18,21]. The fact that these populations are maintained at a near constant ratio within the tissue culture system suggested that there had to be a mechanism to maintain this set point, because otherwise the quiescent CD138- cells should be lost from the culture during passaging. We show that the mature MM cancer cell detected in tissue culture has developmental plasticity and can dedifferentiate back into its own chemoresistant, CD138- CSC progenitor. These newly born CSC regain all of the characteristics of the CSC demonstrating that stemness is a trait that is epigenetically controlled. Thus, instead of a CSC, we suggest that stemness is a dynamic, autoregulated phenotype that can be acquired or lost depending on the interaction with surrounding cells.

This interconversion occurs in the absence of support or accessory cells, suggesting that the MM cells themselves secrete cytokines or factors that mediate this plasticity, and in essence sense and make their own niche. The pure CD138- and C138+ populations secreted MIF-1, although to different levels. The secretion of several other cytokines increased but to a much more modest extent, and by blocking or increasing MIF-1 activity, we were able to show that MIF-1 could alter interconversion, and more importantly chemosensitivity. Blocking MIF-1 drives cells to dedifferentiate into chemoresistant CD138- cells, and the addition of rMIF-1 drives cells to differentiate into chemosensitive CD138+ cells. These cells can be toggled back and forth depending on MIF-1 activity or levels, offering new insight into the epigenetic control of MM growth. Too little MIF-1 and cells dedifferentiate; too much MIF-1 and cells differentiate.

CD138+ cells secreted MIF-1 more rapidly, although secretion at a lower level is still detected in the pure CD138- populations. MIF-1 is known to associate with multiple receptors, including CD74, CXCR2/4/7 and CD44 [27–29], and one differential expression may start the interconversion process. Differential gene expression in the CD138- and CD138+ MM cells has been described further supporting the idea that they represent two different functional populations, and expression of genes associated with stemness, such as NANOG, Wnt, and Hedgehog, are preferentially detected in CD138- precursor cells [30]. Additionally, our study demonstrates that small changes in MIF-1 activity or levels in both cell lines and in primary MM tumor material can shift the set point in one direction or the other. Cytokines are also secreted from the BM microenvironment, and both chemical and physical cues, such as adhesion and oxygen tension, may influence interconversion [31–33]. MIF-1 has emerged as a candidate controlling plasticity and interconversion [33–36], and the logical extension of this work will be to validate this in more physiological *in vivo* settings. Perturbation or modulation of the MIF-1 levels could shift the balance in one direction or the other, and thus represents a viable way to increase the percentage of CD138+, chemosensitive cells. In our preliminary cohort of patients, the addition of 4-IPP to bone marrow derived MM cells increased or maintained the CD138- population in 5 out of 6 patients, suggesting that similar mechanisms are at play *in vivo*. We are intrigued by the observation that some patients had very high CD138- pools at harvest, and this might explain intrinsically different patient responses to chemotherapy. While we examined clinical parameters and outcomes (Fig 7SA), it is difficult to make conclusions from this small sample size. In our tissue culture assay, cells are only viable for a short term, and in the future culturing cells in a 3D matrix will permit more detailed analysis.

Recently, others have reported that MIF-1 can regulate the adhesion of MM tumor cells to BM [37]. Higher levels of MIF-1 expression are seen in MM tumor cells than in normal plasma cells, and loss of MIF-1 reduced MM adhesion to BMSC *in vitro*. While MIF-1 expressing cells formed tumors in the bone when injected into SCID-mice, cells lacking MIF-1 only formed tumors in the abdomen or extramedullary space, suggesting that MIF-1 enhances BM colonization. While these authors did not directly examine interconversion, there was an increase in CD138^-^ cells in the tumors taken from extramedullary space in the MIF-1 KD animals. It is entirely possible that loss of MIF-1 and an increase in CD138- CSCs would affect BM homing and adhesion. They also found that the inhibition of MIF-1 either through KD or 4-IPP treatment caused apoptosis in MM cells after treating with Melphalan in the presence of accessory cells[37]. While the mechanism of action of Melphalan and Bortezomib are very different, our data is in agreement with theirs that altering MIF-1 levels affects chemotherapeutic response, highlighting MIF-1 as an important target.

Our model suggests that when presented with the appropriate environment, any myeloma tumor cell might have the ability to acquire “stemness”, which increases the difficulty in treating a moving target. Our very use of chemotherapy and its change to the microenvironment may permit the reactivation of the chemoresistant clone. If interconversion provides a way to continually renew the chemoresistant CSC population, this would contribute to the eventual relapse seen in this disease, and call for the development of therapies to specifically target plasticity.

## Materials and methods

### Cells and reagents

RPMI 8226, U266-B1, and NCI-H929 were purchased and cultured as described (ATCC). RPMI 8226 cells were plated at a cell density of 250,000 cells/mL; U266-B1, 600,000 cells/mL; and NCI-H929, 450,000 cells/mL. Cells were split every 48 hours. Cytokine Array Panel A, MIF Neutralizing antibody (NAB), recombinant MIF (rMIF), MIF Immunoassay all from (R&D); PD 0332991, CFSE (carboxyfluorescein succinimidyl ester) all from SelleckChem; 4-IPP (4-iodo-6-phenylpyrimidine) (Santa Cruz Biotech); MIF RNeasy mini kit (Qiagen); Verso cDNA kit (Thermo Scientific) and Quanititative PCR using gene-specific primers with Absolute Blue SyBr Green ROX (Thermo Scientific) were used per manufacturer’s instructions.

### Flow cytometry and sorting

Cells were stained with CD138 PE-Cy5 (Beckman Coulter), CD38 PE, Isotype Control IgG1 PE, CXCR4 PE, or CD44 FITC (BD Pharmingen), CD74 FITC (Abcam), and Blimp1 Alexa-Fluor 488 (R&D) by standard procedures. All flow cytometry analysis used the BD FACScan, the BioRad S3 Cell Sorter and data was analyzed using CellQuest software. Cells were stained with Vybrant DyeCycle Green Stain (Invitrogen) and cell cycle analysis was performed using CellQuest software. Freshly sorted populations were also resuspended in methylcellulose (Stem Cell Technologies) at 10^4^ cells/ml as per manufacturer’s instructions. Colonies were counted manually and photographs were taken using a light microscope under 4X magnification. Colonies were measured using the annote tab to compare pixel number to the scale created by the Kodak film software.

### Tracking assay

Cells were treated with 5μM PD 0332991 for 72 h., before washing in fresh media and staining with 5μM CFSE for 10 min. Cells were further stained with CD138 PE-Cy5 and CD38 PE and sorted into CFSE^hi^CD138^+^CD38^+^ and CFSE^hi^CD138^-^CD38^+^ and re-plated as above.

### Cytokine array and analysis

Pure CD138^+^CD38^+^ and CD138^-^CD38^+^ populations were replated in complete media, and harvested at days 1,2, 4 for staining with CD38 PE and CD138 PE-Cy5, while media for each time point was stored at -20°C for later assessment using the Human Cytokine Array Panel A (R&D). Capture antibodies against the cytokines listed in Fig 4A were spotted in duplicate on nitrocellulose membranes. Cell culture supernatant is mixed with a cocktail of biotinylated detection antibodies, and the sample/antibody mixture is then incubated with the array. Any cytokine/detection antibody complex present is bound by its cognate immobilized capture antibody on the membrane. Arrays were analyzed using IRDye 800CW Streptavidin (LICOR) and the LICOR Imaging System following vendor’s instructions. Raw values for each cytokine detected in media alone were subtracted from values for each cytokine at each time point. Data presented is representive of N = 2 experiments.

### Chemosensitivity assay

Sorted populations were treated with 0.01μM Bortezomib (SelleckChem) for 24 h. Chemosensitivity was measured as the change in viability before and after treatment as measured by trypan blue exclusion. MTT Assay (Promega) used to measure viability as described.

### Primary MM patient samples

All research involving human participants was approved by the authors' Institutional Review Board (IRB) of SUNY Downstate Medical Center, and all clinical investigation was conducted according to the principles expressed in the Declaration of Helsinki. Informed consent, written or oral, was obtained from all participants. Bone marrow aspirates from MM patients at Kings County Hospital or University, obtained with informed consent, were purified by passage through the MM enrichment kit (RosetteStep). RosetteStep MM removes cells with CD2, CD14, CD33, CD41, CD45RA, CD66b and glycophorin A on red blood cells (RBCs). When centrifuged over a buoyant density medium, the unwanted cells pellet along with the RBCs. The purified multiple myeloma cells are present as a highly enriched population at the interface between the plasma and the buoyant density medium. Purified plasma cells were either grown in tissue culture +/- 4-IPP or immediately fixed and stained with CD38 PE and CD138 PE-Cy5. Viability of cells was checked by trypan blue staining. Cells were grown using the patient’s own serum, obtained from peripheral blood.

### Statistics

All data are shown as the mean ± SD. The statistical analyses were performed using the Exact Mann-Whitney 2-sided tests, a semi-parametric (SAS PROC MULTTEST with bootstrapping) approach was used to (1) apply a t-test of monotonic trend, allowing for possible non-normality; (2) correct p-values derived from pair-wise t-tests for possible non-normality and for multiple testing. The Satterthwaite adjustment for heteroscedasticity was also applied. Selected pair-wise comparisons were made using t-tests (with Satterthwaite corrections to allow for possible unequal variances). The distribution of the dependent variable was checked for symmetry and for the presence of outliers. All other statistics used Student t-test (2 tail).

## Supporting information

S1 Fig Purification of CD138- and CD138+ populations. **A)** Dot plot of unsorted RPMI8266 cells **B)** Dot Plot of cells stained with CD138 and CD38. Three populations are visible. C) Viablity measured by trypan blue staining of population i-iii. The CD138^null^ population (bottom in the dot plot) was non-viable and was gated out of all future analysis. D) Gated CD138- (ii) and CD138+ (iii) populations, sorted to purity (E). **F)** Viability measured by MTT assay of sorted CD138- RPMI8226 cells 24 and 48 h post sort and replating. **G)** Viability measured by MTT assay of sorted CD138+ RPMI8226 cells 24 and 48 h post sort and replating. No significant loss in viability after 48 h was observed for either sorted population.
(JPG)

S2 Fig **Cell proliferation and viability of sorted populations of RPMI8226 (A), U266-B1 (B), and NCI-H929 (C). A,B, C)** CD138+ (solid) or CD138- (dashed) cells were replated and counted at days 5, 10 and 13 post sort. **D-I)** CD138+ (left) or CD138- (right) cells were replated and viability measured by trypan exclusion at days 0, 5, 10, and 13 post sort. RPMI8226 (D-E), U266-B1 (F-G), and NCI-H929 (H-I). Each sorted population is proliferating from day 0 to day 13 and there is no significant change or loss in viability between CD138- and CD138+ populations for all three MM cell lines. **J)** Histogram of unsorted U266-B1 MM stained with CD138. **K)** Dot Plot of Sorted CD138+ and CD138- U266-B1 cells. The CD138null population (left in the dot plot) was non-viable and was gated out of all analysis. **L,M)** Sorted populations of CD138- and CD138+ cells. **N)** Histogram of unsorted NCI-H929 cells stained with CD138. **O)** Dot Plot of Sorted CD138+ and CD138- NCI-H929 cells. The CD138null population (bottom in the dot plot) was non-viable and was gated out of all analysis. **P, Q)** Sorted populations of CD138- and CD138+ cells. **R)** Cell counts for experiment the plated, pure, sorted CD138- and CD138+ population. Growth rates were calculated and are the mean of the growth seen over a 5 day period (1.1 for CD138- and 1.2 for CD138+). **S)** Cell counts plotted. **T)** CD138- plated experiment. 250000 cells were plated at day 0. 0.73% of 250,000 is 1825 contaminating CD138+ cells. We predicted that this population would expand to 2190 cells at day 2, given the growth rate of 1.2 seen for these cells. However, we detected 76,480 CD138+ cells or 23.9% of the total population of 320,000 cells. **U)** CD138+ plated experiment. 250,000 cells were plated at day 0. 0.17% of 250,000 is 425 contaminating CD138- cells. We predicted that this population would expand to 466 cells at day 2, given the growth rate of 1.1 seen for these cells. However, we detected 13,200 CD138- cells or 3.3% of the total population of 400,000 cells.
(JPG)

S3 Fig Sorting profile. Cells were gated for FSC and SSC. CD138 and CD38 co-staining revealed three populations, which were tested for viability by trypan blue staining. Population iii was non-viable and excluded from all future analysis. Population i and ii were then sorted to >98% purity.
(JPG)

S4 Fig Raw values obtained by LICOR imaging system for cytokine arrays at each time point for both CD138- (A) and CD138+ (B) and media alone.
(JPG)

S5 Fig Clinical descriptors of patients in MM cohort. (PDF)
