# Supplementary figures and images for: Macrophage Inhibitory Factor-1 (MIF-1) controls the plasticity of multiple myeloma tumor cells
Source: PLoS One. 2018 Nov 1;13(11):e0206368. doi: 10.1371/journal.pone.0206368 (PMC6211687; doi:10.1371/journal.pone.0206368)

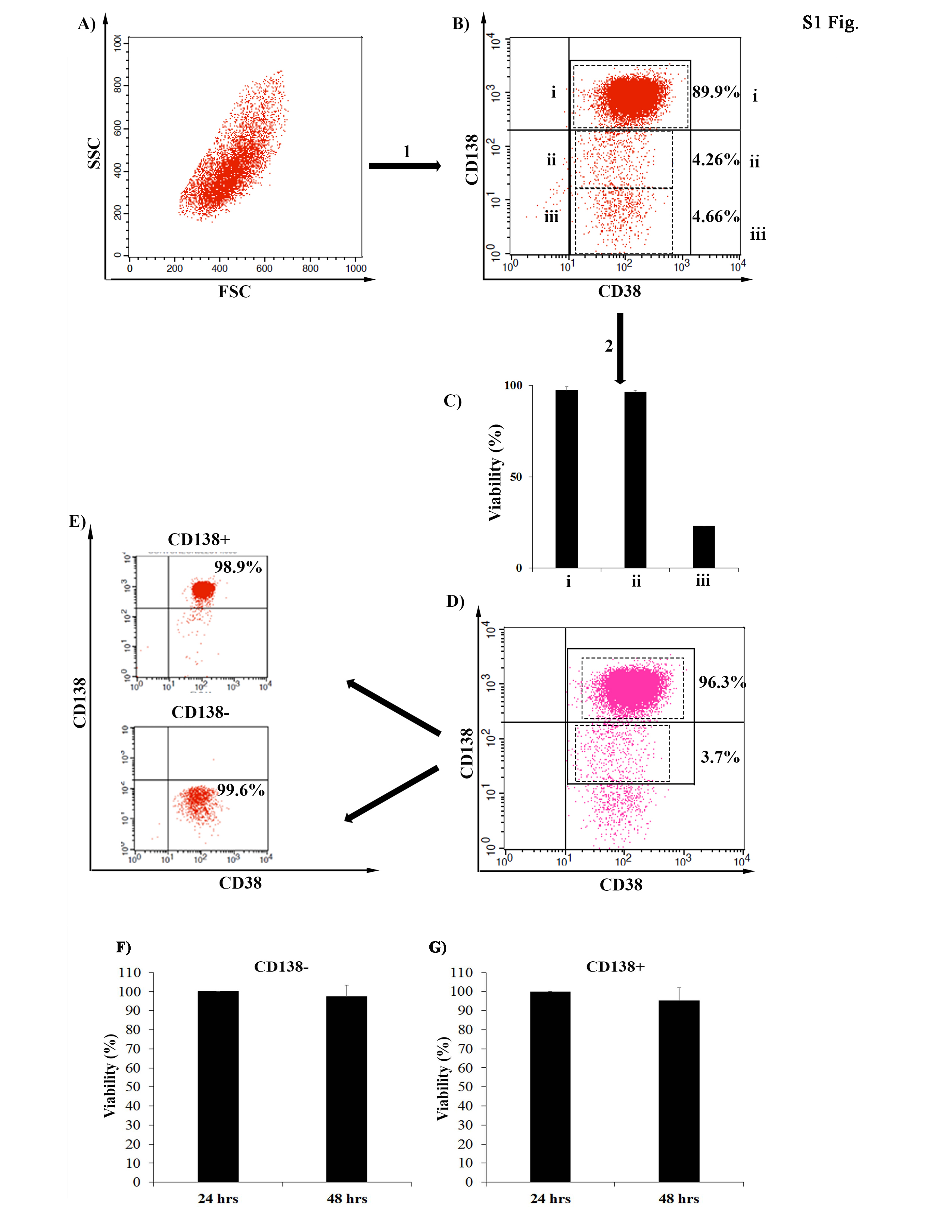

Supplement: S1 Fig — A) Dot plot of unsorted RPMI8266 cells B) Dot Plot of cells stained with CD138 and CD38. Three populations are visible. C) Viablity measured by trypan blue staining of population i-iii. The CD138null population (bottom in the dot plot) was non-viable and was gated out of all future analysis. D) Gated CD138- (ii) and CD138+ (iii) populations, sorted to purity (E). F) Viability measured by MTT assay of sorted CD138- RPMI8226 cells 24 and 48 h post sort and replating. G) Viability measured by MTT assay of sorted CD138+ RPMI8226 cells 24 and 48 h post sort and replating. No significant loss in viability after 48 h was observed for either sorted population. (JPG) [file pone.0206368.s001.jpg]

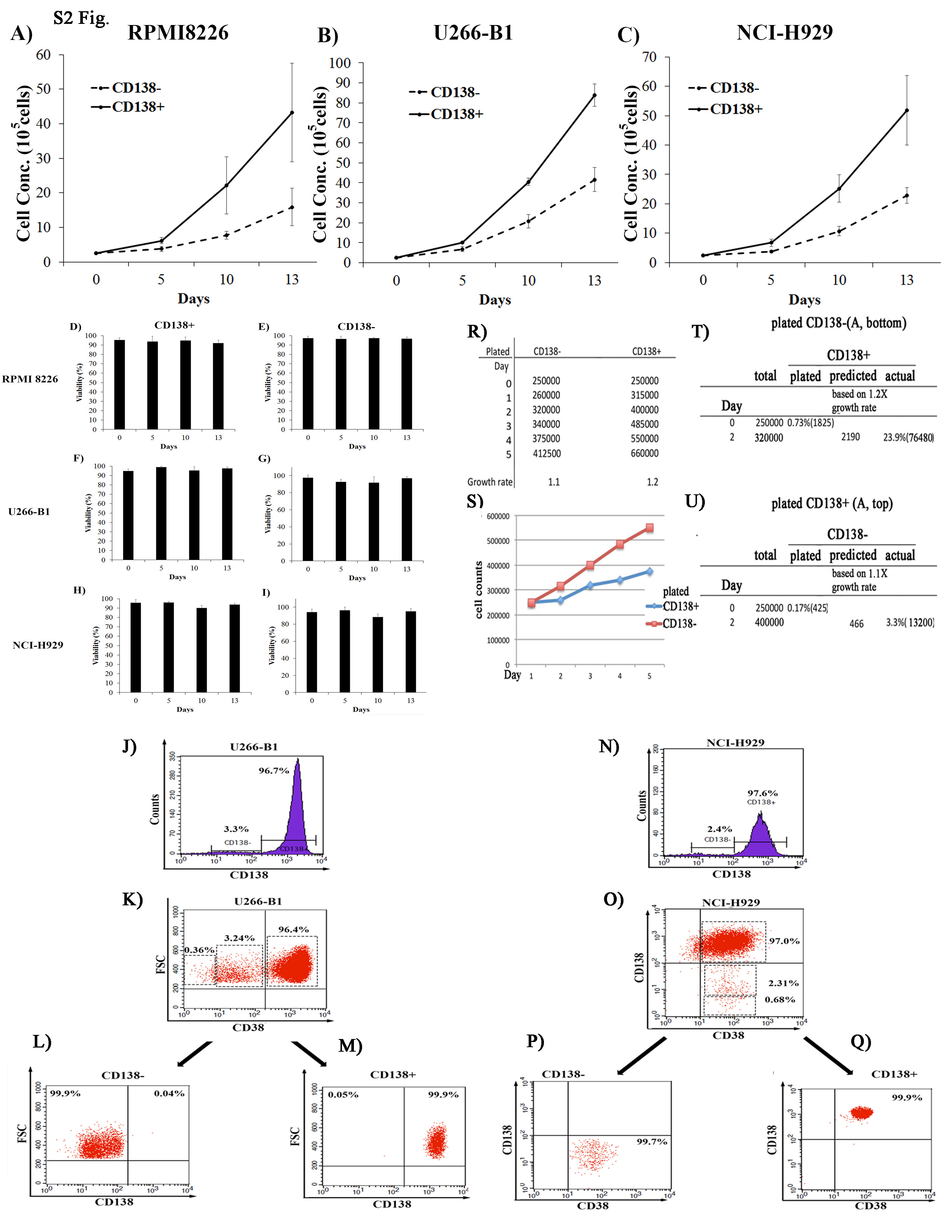

Supplement: S2 Fig — Cell proliferation and viability of sorted populations of RPMI8226 (A), U266-B1 (B), and NCI-H929 (C). A,B, C) CD138+ (solid) or CD138- (dashed) cells were replated and counted at days 5, 10 and 13 post sort. D-I) CD138+ (left) or CD138- (right) cells were replated and viability measured by trypan exclusion at days 0, 5, 10, and 13 post sort. RPMI8226 (D-E), U266-B1 (F-G), and NCI-H929 (H-I). Each sorted population is proliferating from day 0 to day 13 and there is no significant change or loss in viability between CD138- and CD138+ populations for all three MM cell lines. J) Histogram of unsorted U266-B1 MM stained with CD138. K) Dot Plot of Sorted CD138+ and CD138- U266-B1 cells. The CD138null population (left in the dot plot) was non-viable and was gated out of all analysis. L,M) Sorted populations of CD138- and CD138+ cells. N) Histogram of unsorted NCI-H929 cells stained with CD138. O) Dot Plot of Sorted CD138+ and CD138- NCI-H929 cells. The CD138null population (bottom in the dot plot) was non-viable and was gated out of all analysis. P, Q) Sorted populations of CD138- and CD138+ cells. R) Cell counts for experiment the plated, pure, sorted CD138- and CD138+ population. Growth rates were calculated and are the mean of the growth seen over a 5 day period (1.1 for CD138- and 1.2 for CD138+). S) Cell counts plotted. T) CD138- plated experiment. 250000 cells were plated at day 0. 0.73% of 250,000 is 1825 contaminating CD138+ cells. We predicted that this population would expand to 2190 cells at day 2, given the growth rate of 1.2 seen for these cells. However, we detected 76,480 CD138+ cells or 23.9% of the total population of 320,000 cells. U) CD138+ plated experiment. 250,000 cells were plated at day 0. 0.17% of 250,000 is 425 contaminating CD138- cells. We predicted that this population would expand to 466 cells at day 2, given the growth rate of 1.1 seen for these cells. However, we detected 13,200 CD138- cells or 3.3% of the total population of 400,000 cells. [file pone.0206368.s002.jpg]

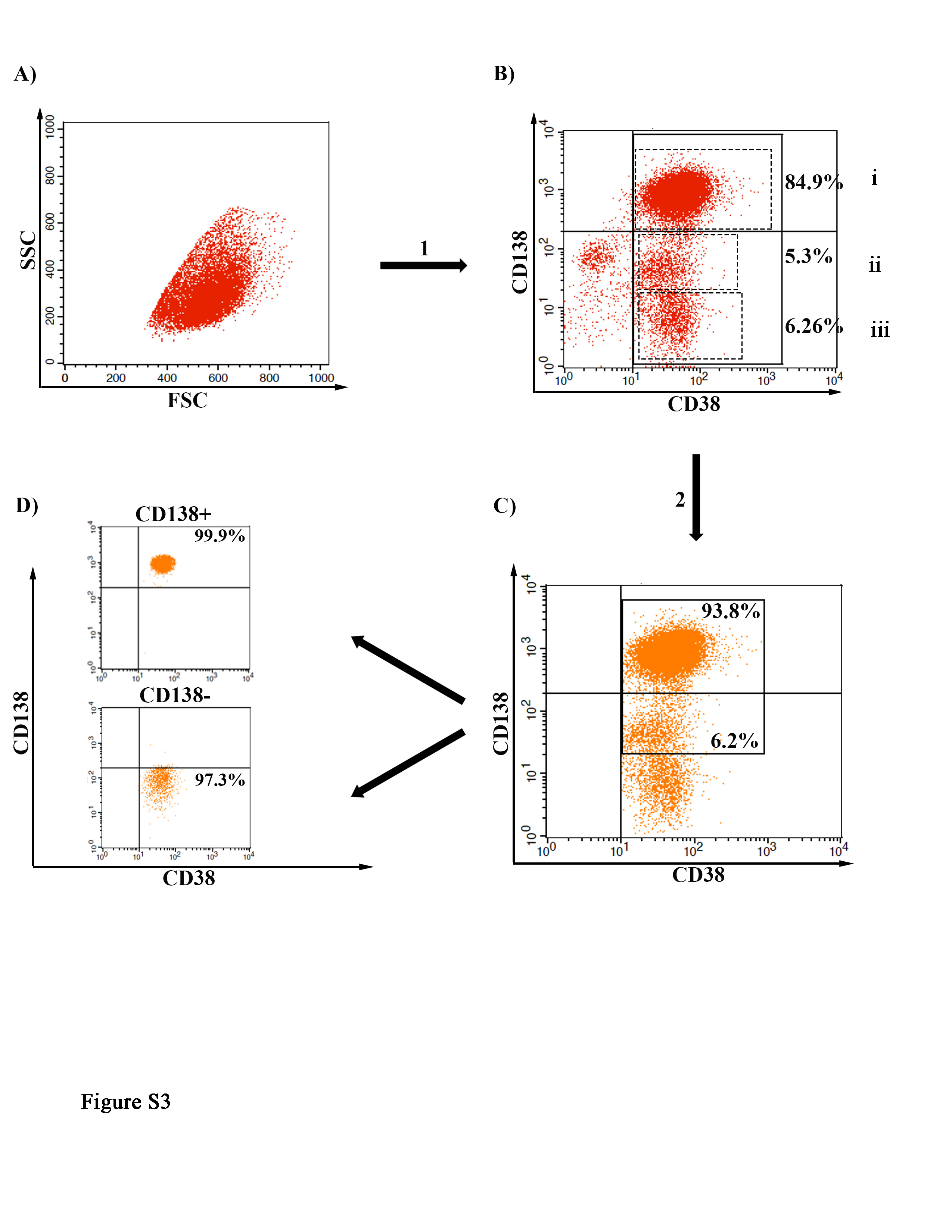

Supplement: S3 Fig — Cells were gated for FSC and SSC. CD138 and CD38 co-staining revealed three populations, which were tested for viability by trypan blue staining. Population iii was non-viable and excluded from all future analysis. Population i and ii were then sorted to >98% purity. (JPG) [file pone.0206368.s003.jpg]

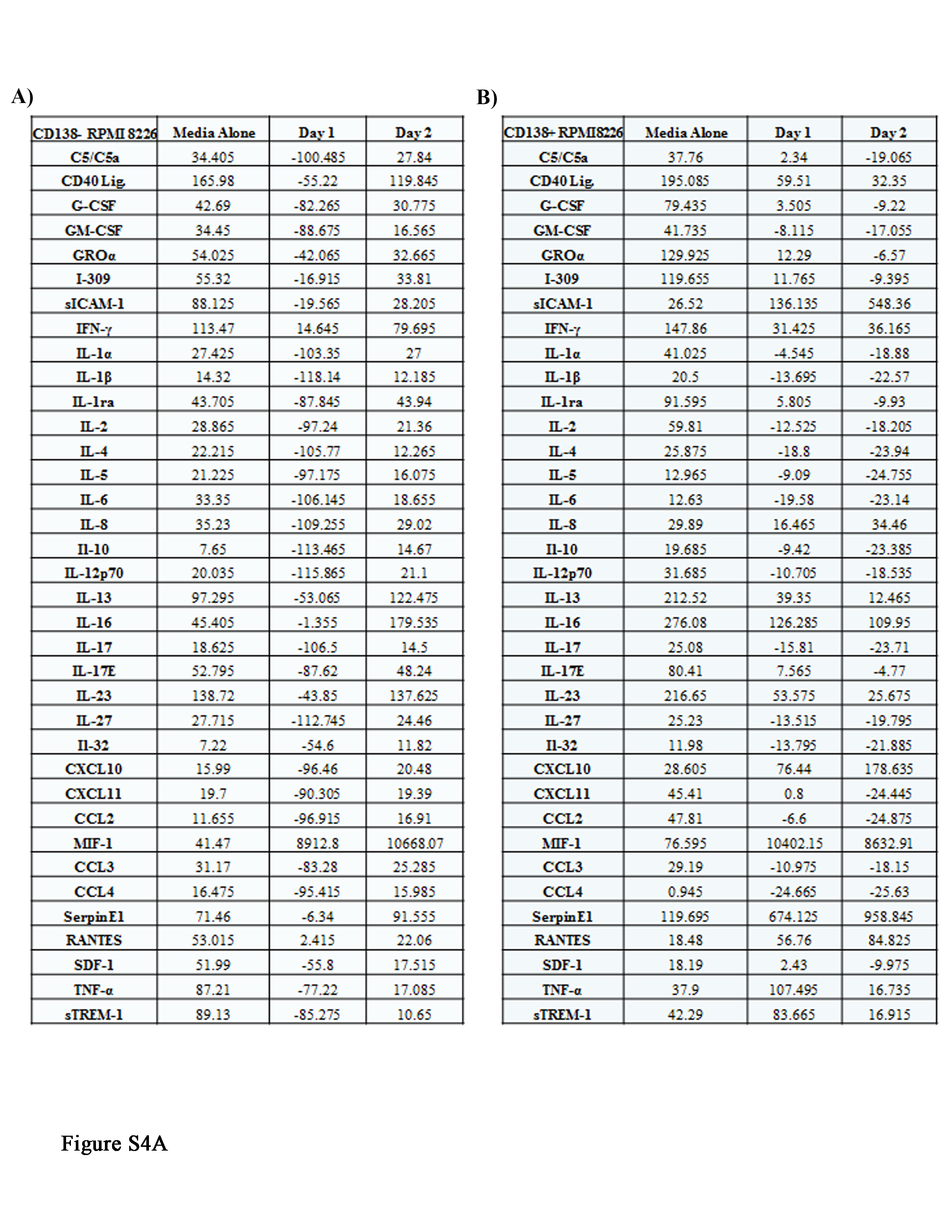

Supplement: S4 Fig — Raw values obtained by LICOR imaging system for cytokine arrays at each time point for both CD138- (A) and CD138+ (B) and media alone. (JPG) [file pone.0206368.s004.jpg]
