# Supplementary material for: Macrophage Inhibitory Factor-1 (MIF-1) controls the plasticity of multiple myeloma tumor cells
Source: PLoS One. 2018 Nov 1;13(11):e0206368. doi: 10.1371/journal.pone.0206368 (PMC6211687; doi:10.1371/journal.pone.0206368)
Supplement: S5 Fig — (PDF) [file pone.0206368.s005.pdf]

Patient	age	Hgb g/dl	Ca mg/dl	Bone Disease	Renal disease (Cr) mg/dl	M spike g/dl	M protein	cytogenetics	% plasma cells in BM	Response To 4 cycles Btz + dex
B	63	8.4	10.0	+	1.25	5.3	IgG L	normal	20-30%	PR
E	86	6.2	12.7	-	1.71	5.3	IgA L	Trisomy 11; gain 1q21	100%	CR
F	44	8.5	7.6	+	0.98	6.8	IgG L	add 9,19	80%	VGPR
H	64	8	12.4	+	12.84	2.1	IgG K	t(11;14), add 15	80%	SD
I	74	9.2	9.2	+	1.23	1.7	IgG L	Hyperdyploid; add 1,7, 9,13, 15;<br>4 copies of genes on 4, 11, 14, 16, 17, gain of Q21	70%	PR
A	68	8.1	10.7	+	0.84	6.7	IgG K	Normal	70%	MR

MR: Minimal response
CR: complete response
PR: partial response
VGPR: Very Good Partial Response
SD: stable disease

BTZ: Bortezomib
Dex: dexamethasome

Figure S5
